# Comparative Study of the Resistance of Six Hawaii-Grown Bamboo Species to Attack by the Subterranean Termites *Coptotermes formosanus* Shiraki and *Coptotermes gestroi* (Wasmann) (Blattodea: Rhinotermitidae)

**DOI:** 10.3390/insects2040475

**Published:** 2011-11-03

**Authors:** Nirmala K. Hapukotuwa, J. Kenneth Grace

**Affiliations:** College of Tropical Agriculture & Human Resources, University of Hawaii at Manoa, 3050 Maile Way, Gilmore Hall 310, Honolulu, HI 96822, USA; E-Mail: kennethg@hawaii.edu

**Keywords:** *Coptotermes formosanus*, *Coptotermes gestroi*, bamboo, wood resistance

## Abstract

Bamboo is widely grown and utilized as a construction material around the world, particularly in the tropics. At present, there are about 70 bamboo species and varieties recorded from Hawaii. The objective of our study was to determine the relative resistance of six Hawaii-grown bamboo species to attack by *Coptotermes formosanus* Shiraki and *Coptotermes gestroi* (Wasmann). Four-week laboratory feeding trials were performed as described in standard E1-09 of the American Wood Protection Association (AWPA 2009). Samples of each of the six bamboo species were individually exposed to 200 termites (with 10% soldiers); and termite mortality, wood mass loss, and visual appearance of the samples (on a scale of 0–10) were recorded at the conclusion of the trail. Mean mass losses of the six species as a result of termite feeding ranged from 13–29%; with the two most resistant bamboo species, *Gigantocholoa pseudoarundinacea* and *Bambusa oldhamii*, demonstrating significantly greater resistance to termite attack than the most susceptible bamboo species, *Guadua anguistifolia*, with both termite species. *Dendrocalamus brandisii*, *Dendrocalamus latiflorus*, and *Bambusa hirose* were intermediate in their termite resistance. Overall, we observed very little difference in wood preference between *C. formosanus* and *C. gestroi*. Although bamboo is a very promising construction material, and species clearly differ in their susceptibility to termite attack, all six species evaluated in the present study would require additional protection for use under conditions of high termite pressure.

## 1. Introduction

Bamboos are one of the most useful natural resources in many parts of the world. Due to their various properties they have been named as the most important sustainable and environmentally helpful crop on the planet [1]. Presently there are about 1575 accepted bamboo species plus several other species with incorrect names [2]. They are naturally distributed in all continents except Europe and Antarctica. Bamboos are gaining popularity worldwide for ornamental and economic purposes [3]. Asia is the continent where bamboo is most integrated into the culture. Bamboos are widely used for house constructions in earthquake-prone areas, especially in China, India, Japan, Malaysia, Indonesia and the Philippines [4]. Interestingly, in many Asian countries, the use of bamboo is declining because the resource is being overused due to urbanization and increasing population [5]. The Americas (North, Central and South), Africa (Tropical, South and Madagascar), Australia (especially Northern Australia), and the Pacific (New Guinea, Pacific and Polynesian) also contain many different bamboo species. They have cultural, construction, and historical value. Some of the main usages are as constructional materials, bridges, fencing, for basket making, furniture, mats, tool handles, musical instruments, paper and pulp making and food for humans and livestock [4,6]. Young bamboo shoots are a very popular food worldwide, including the USA , where currently more than 30,000 tons of edible shoots are consumed each year [5]. Also, some bamboo species are grown for ecological purposes such as stabilization and erosion prevention. Bamboos grow mainly in tropical areas with a few species found in the subtropical and temperate regions.

Tropical islands such as Hawaii provide ideal habitats for bamboos. Two species, *Bambusa vulgaris* and *Schizostachyam glaucifolium*, are linked with ancient Polynesian traditions [3]. The Polynesians brought these two species during their oceanic navigation. These Polynesian bamboos are apparently native to Fiji [7]. There are about 70 species and varieties recorded from Hawaii [3]. All are introduced; some are available in large numbers whereas others are limited to few local nurseries. Bamboos are mainly distributed on the islands of Hawaii (Big Island), Oahu, and Maui and there are many organizations/private companies that make use of bamboo. Bamboo is known as “Ohe” in Hawaiian. Hawaiians use bamboo for different purposes, mainly for construction, but in addition for furniture, musical instruments (the three holed nose flute “ohe hano ihu”), fences, mats, utensils, agricultural tools, ladders, ornaments, toys, fishing tools, and food (immature young shoots). Also, some people in Hawaii plant bamboos for erosion prevention.

Much research has been carried out on wood resistance against termites using different timbers but very limited research has been done on bamboos. Mishra and Rana [8] and Mishra and Thakur [9] conducted laboratory evaluations of the natural resistance of different bamboo species to termites in India. Using *Microcerotermes beesoni* and 13 bamboo species found in India, they found that natural resistance of bamboos was more or less comparable to that of some of the moderately durable commercially important timber species. Furthermore, they reported that the outer layer of bamboo is highly resistant and that termites normally invade bamboo from the cut end portion only. Dhawan *et al.* [10] studied termite damage in relation to the chemical composition of bamboos. These authors found that nitrogen content in bamboo was directly related to termite damage. The quantity of lignin, ash, and silica present in bamboo influenced termite damage and played a significant role in termite resistance. Gogoi and Sonowal [11] did an experiment using *Bambusa tulda* to test the termite and fungal resistance of chemically treated bamboo. They found that dithiocarbamate and its copper complex was a good cellulase inhibitor. All treated bamboo samples had less weight loss than untreated samples. Dhawan and Mishra [12] performed another study on the influence of felling season and moon phase on the natural resistance of bamboos against termites. They found bamboos felled during moon phase were less resistant to termites than those felled in dark phase. In addition, Higuchi [6] analyzed the chemistry and biochemistry of three different bamboo species common in Japan; Sanyal *et al.* [13] wrote a review on strength properties and uses of bamboos in India; and Mishra [14] studied the structural use of bamboo in rural housing in India.

In the present study we examined the resistance of six bamboo species grown on Maui, Hawaii to attack by *Coptotermes formosanus* and *C. gestroi*. These findings will be helpful both to identify termite-resistant species that could be grown locally in Hawaii, and to determine which commercial species will require preservative treatment before use in regions with high termite hazard.

## 2. Materials and Methods

A no-choice, or single choice, test, described as a standard method in Standard E1-09 of the American Wood Protection Association (AWPA 2009), was used to assess the levels of resistance to termite attack of six different bamboo species grown on Maui, Hawaii, namely *Bambusa hirose* (BH) (Hirose's bamboo), *Bambusa oldhamii* (BO) (Oldhami bamboo/giant timber bamboo), *Dendrocalamus brandisii* (DB) (sweet dragon bamboo), *Dendrocalamus latiflorus* (DL) (sweet giant bamboo), *Gigantocholoa pseudoarundinaceae* (GP) (great giant bamboo), and *Guadua angustifolia* (GA) (“guadua”). All bamboo samples were provided by Whispering Winds Bamboo, Hana, Maui.

### 2.1. Apparatus and Materials

#### 2.1.1. Bamboo Samples

Samples were cut from six species of bamboo using a band saw. Although the outer wall of the samples varied in thickness, samples were cut to include both outer and inner surfaces, and each test sample was approximately 25 mm (1 inch) by 25 mm (1 inch) by 6 mm (1/4 inch). All samples were autoclaved (Getinge Auto Clave, Gettings USA, Inc, New York) at 256 °C and 20 PSI for 60 min to remove molds. For each bamboo species there were five replicates and three environmental controls (exposed to the same test conditions, but without termites). All samples were dried in a drying oven (calibrated with a Salvis thermometer) at 90 °C for 24 h and allowed to cool to room temperature in a desiccator for one hour. Dry samples were weighed using a Mettler AE 163 balance.

### 2.2. Experimental Design

The test containers (jars) were 85 mm diameter and 97 mm tall, made of polystyrene, and with a plastic screw top lid. Two sets of jars were used: test jars (with live termites) and environmental control jars (without termites). Each jar contained 150 g of silica sand [Fine granules (40–100 mesh, Fisher Scientific, Fair Lawn, NY, USA)], 30 mL of distilled water (to hold moisture), and a single bamboo wafer.

### 2.3. Bioassays and Termites

Termites were collected from two different field sites: *C. gestroi* from Kalaeloa (formerly Barber's Point Naval Air Station) on the southwest side of the Island of Oahu, Hawaii, and *C. formosanus* from colonies located on the Manoa campus of the University of Hawaii. Termites were collected using techniques modified from those of [15] and [16]. Two hundred live termites (180:20, workers:soldiers) were placed into each test container. The jar tops were replaced loosely. The jars were placed in an unlighted incubator at 28 °C and 72–80% RH for four weeks. Every week, all jars were visually inspected and tunneling patterns and termite activities were recorded.

At the end of the four-week test period, all jars were disassembled and the wafers were removed. Live termites were counted to record their mortality rates. All wafers were allowed to air dry at room temperature for 24 h, then oven dried at 90 °C for 24 h and allowed to cool to room temperature in a dessicator for one hour. Finally, all waifers were reweighed to determine the amount consumed by termites, and also visually rated using the scale described in AWPA (2009) Standard E1-09 (see Tables 1 and 2).

To compare feeding rates on the six different bamboo species between *C. formosanus* and *C. gestroi*, we used one-way ANOVA and TUKEY HSD for means separation (SAS 9.2). Also, two-way ANOVA and the Ryan-Einot-Gabriel-Welsch Multiple Range Test [REGWQ] were done using SAS 9.2 to detect any significant differences in mean mass loss among the six bamboo species, as well as between the two termite species (see Table 3).

## 2. Results and Discussion

**Figure 1:**
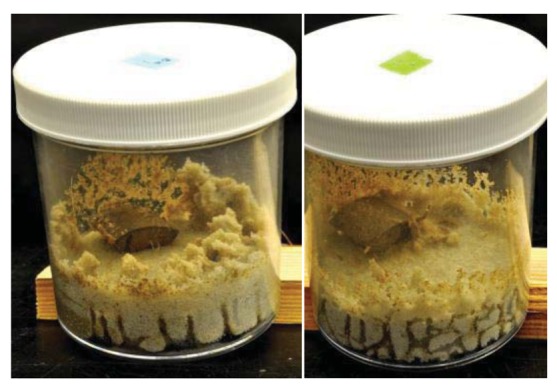
Sample test jars of *C. formosanus* (**left**) and *C. gestroi* (**right**).

**Figure 2:**
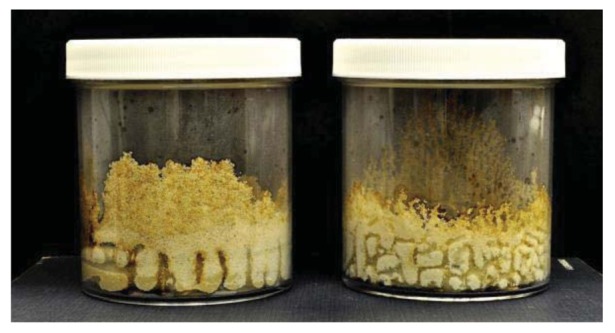
Tunneling patterns of *C. formosanus* (**left**) and *C. gestroi* (**right**).

As has been previously noted by Grace *et al.* [17], we observed some differences between *C. formosanus* and *C. gestroi* in tunneling patterns (Figures 1 and 2). *Coptotermes gestroi* made a greater number of narrow and highly branched tunnels, while *C. formosanus* made fewer, and less branched tunnels. In addition, *C. gestroi* constructed tunnels all the way to top of all jars within first three weeks; but *C. formosanus* made very few tunnels to the top of only a few jars within this same time period. Within the first two weeks, both termite species were very active in tunneling and moved onto the bamboo wafers. During the first week of observations, *C. gestroi* showed very light feeding on *Bambusa hirose*, *B. oldhamii*, *D. latiflorus*, and *Guadua angustifolia*; whereas *C. formosanus* caused no visible damage to any bamboo wafer. During the second week of inspection, *C. gestroi* did heavy damage to *B. oldhamii* and *Guadua angustifolia* However, *C. formosanus* exhibited light damage to all the bamboo types, except *D. latiflorus* and *Dendrocalamus brandisii* which had moderate damage. Since some of the wafers with both termite species were covered with sand, we had some difficulty in visually estimating feeding rates. During the third and fourth weeks, both termite species exhibited less tunneling activity but relatively high feeding activity. *Coptotermes formosanus* in particular showed heavy feeding on *Bambusa hirose* and *Guadua angustifolia*. Also, we observed that all of our test samples were invaded by the termites from the cut sides and through the inner layer, rather than directly through the exterior surface. The reason for this pattern of attack may be that the outer layer of bamboo has a considerable amount of ash and silica [18,19], and that these compounds help in improving natural durability as well as in imparting strength to bamboos [13].

**Figure 3:**
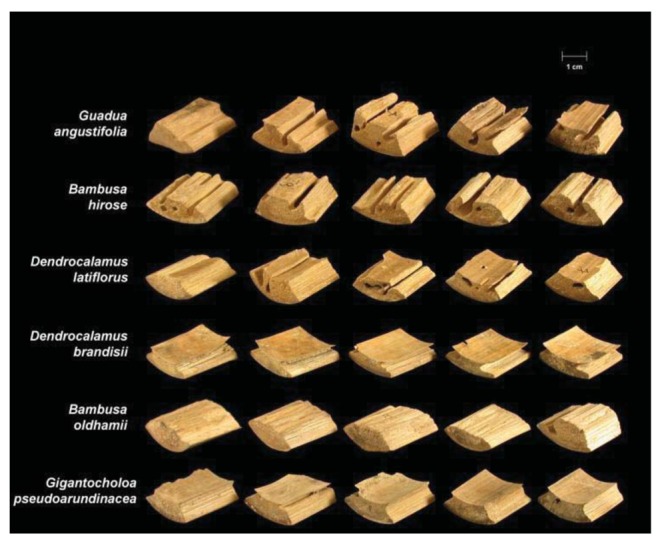
Bamboo blocks showing damage from *C. formosanus*. Image courtesy of Robert Oshiro.

**Figure 4:**
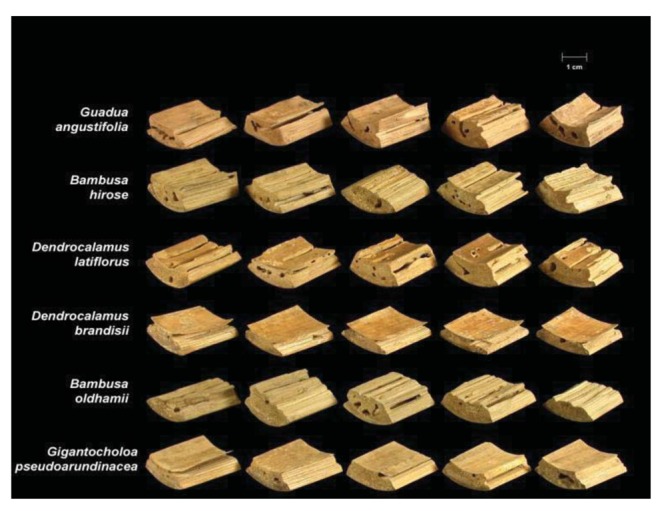
Bamboo blocks showing damage from *C. gestroi*. Image courtesy of Robert Oshiro

Termite attack on the six bamboo species after four weeks is depicted in Figures 3 and 4. Summaries of the results of our data analyses are presented in Tables 1 and 2. Mean visual ratings of termite damage ranged from moderate to severe with both termite species. Among the six species of bamboo tested, overall mass losses from both termite species ranged from 13%–29%. Maximum damage was observed on *Guadua angustifolia* (GA) for both termite species (*C. formosanus* 28.84%, *C. gestroi* 24.52%) and minimum damage was observed in *Gigantocholoa pseudoarundinacea* (GP) (*C. formosanus* 14.20%, *C. gestroi* 12.96%). The remaining bamboo species showed intermediate mass loss values. Damage on each bamboo species was similar from both termites When we compared termite feeding on these bamboo species to results obtained previously with three commercial woods (Douglas fir, southern yellow pine and redwood), *C. formosanus* showed greater feeding on Douglas fir (33.67 ± 7.85) and southern yellow pine (27.98 ± 10.63) than on the six bamboo species. However, with *C. gestroi*, feeding on bamboo was greater than that observed on to Douglas fir (13.39 ± 9.52) or southern yellow pine (13.85 ± 9.35). Both termite species fed least on redwood in comparison to either other commercial wood or bamboo (*C. formosanus* − 4.75 ± 2.73, *C. gestroi* − 6.28 ± 4.78).

Two way ANOVA indicated significant differences in mean mass loss values among the six different bamboo species (*F* = 20.53, *DF* = 5, *P* < 0.0001), but no significant difference in feeding between the two termite species (*F* = 3.08, *DF* = 1, *P* = 0.0855.This suggests that both termite species have similar preferences for bamboo.

**Figure 5:**
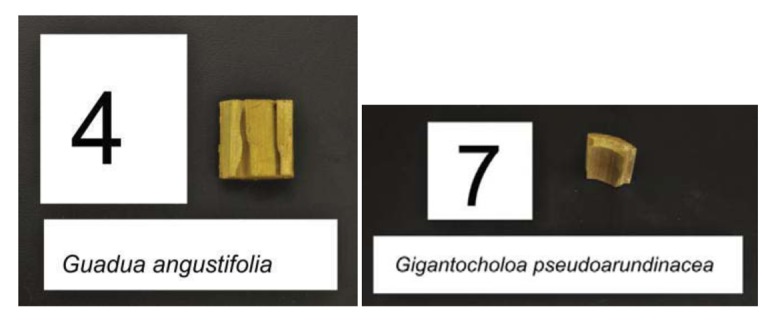
Sample visual ratings for *C. formosanus*.

Mean percentage termite mortality differed significantly both between termite species (*F* = 9.26, *Df* = 1, *P* = 0.0038) and among the six different bamboo species (*F* = 12.07, *Df* = 5, *P* < 0.0001) (Figure 5). Compared to *C. formosanus*, *C. gestroi* showed higher mortality, possibly due the test conditions being more favorable for the subtropical *C. formosanus*.

According to the wood durability classification developed by Grace *et al.* [20], the six bamboo species can be categorized in Table 4 for both termite species. Using to this classification, all bamboo species tested are not very resistant to either termite species. This supports the opinion of Mishra and Rana [8] that bamboos should be considered perishable timbers and are not generally resistant to termite attack. Different feeding on different bamboo species may be due to some differences in chemical composition. For example, a higher quantity of carbohydrates (especially starch content) can make the timbers relatively more susceptible to insect attack [21–23]. Dharwan *et al.* [10] also found, however, that oligosaccharides and polysaccharides do not play a significant role in termite resistance. However, lignin, nitrogen, ash, and silica content may have an effect on termite resistance. For example, the quantity of lignin present in bamboo has been noted Dhawan *et al.* [10] to be inversely related to termite damage. Therefore high lignin content leads to low termite damage; and lignin, interferes with digestion by binding both carbohydrate substrate and digestive enzymes in the insect gut [10]. High nitrogen content (nitrogen rich food) is preferred by termites, and the nitrogen content in bamboo may be directly related to termite damage. Higher ash content is not preferred by termites, is not absorbed in their body and passes through in the feces. Some of the minerals found in woods and bamboos may also have some toxic effect or disturb the insect's physiology. In addition, the presence of crystal from silica in bamboo inhibits digestion and has been termed a digestibility reducer [10]. Thus, the silica content of bamboo may be inversely related to termite damage.

Dhawan and Mishra [12] noted that seasonal variation in bamboo growth or harvest may also have some effect on termite resistance, possibly due to changes in chemical composition within the bamboo species. The carbohydrate content (free sugars and carbohydrates) increases increased during the summer. As a result, termite feeding rates may also increase. However, during the winter, carbohydrate content is low and bamboo growth rate is also low. Therefore, termites do not prefer to feed on bamboo during this period. Dhawan and Mishra [12] found that bamboos harvested during winter months were more resistant to termite attack than those harvested during summer. Some phenolic compounds have also been shown to contribute to increased resistance to termites [24].

Shukla *et al.* [25] found that bamboos are susceptible to a large number of disease-causing fungi, and fungus infected wood can be attractive to termites. Therefore fungi have been considered the primary invader in bamboo, followed by termites [9]. Finally, it is possible that characteristics of bamboo such as age, diameter, height, felling season, seasoning method, *etc.* may also have an effect on termite attack [9].

## 4. Conclusions

*Coptotermes formosanus* and *C. gestroi* show very similar preferences for six different bamboo species grown in Hawaii. Our findings provide evidence of the relative resistance levels of these bamboos, and it is important to note that none of them were highly resistant to termite attack and most should be considered perishable. In further work, we intend to explore both additional Hawaii-grown bamboo species that may show greater termite resistance, and use of disodium octaborate tetrahydrate and other preservatives to protect susceptible bamboo species.

## Figures and Tables

**Table 1:** Summary of results for *C. formosanus* from no-choice test. (^a^ Values in parentheses are standard deviations; means within a column followed by the same letter are not significant at the 5% level (one way ANOVA, Tukey's HSD—SAS 9.2), Rating: 10 (sound), 9.5 (trace, surface nibbles permitted), 9 (slight attack up to 3% of cross sectional area affected), 8 (moderate attack, 3–10% of cross sectional area affected), 7 (moderate/severe attack, penetration, 10–30% of cross sectional area affected), 6 (severe attack, 30–50% of cross sectional area affected), 4 (very severe attack, 50–70% of cross sectional area affected) or 0 (failure)).

**Bamboo Species**	**Mean Visual Rating**	**Mean Mass Loss (g)**	**Mean Percent Mass loss (%)**	**Mean Percent Termite Mortality (%)**
*Guadua angustifolia* (GA)	5.20	0.6912	28.84	18.90
	(±1.10)	(±0.11) a	(±2.12)	(±5.67)cd
*Bambusa hirose* (BH)	6.00	0.6214	24.98	24.90
	(±1.41)	(±0.07) a	(±4.11)	(±3.34)abc
*Dendrocalamus latiflorus* (DL)	7.00	0.5744	21.12	25.40
	(±0.00)	(±0.05)ab	(±2.63)	(±6.55)bd
*Dendrocalamus brandisii* (DB)	6.00	0.5227	19.68	30.10
	(±0.00)	(±0.07)ab	(±3.95)	(±12.53)bd
*Bambusa oldhamii* (BO)	6.40	0.4838	18.08	38.10
	(±0.55)	(±0.07)b	(±4.16)	(±6.54)b
*Gigantocholoa pseudoarundinacea* (GP)	7.40	0.4300	14.20	32.30
	(±0.55)	(±0.03)b	(±0.95)	(±5.03)bd

**Table 2:** Summary of results for *C. gestroi* from no-choice test. (^a^ Values in parentheses are standard deviations; means within a column followed by the same letter are not significant at the 5% level (one way ANOVA, Tukey's HSD—SAS 9.2), Rating: 10 (sound), 9.5 (trace, surface nibbles permitted), 9 (slight attack up to 3% of cross sectional area affected), 8 (moderate attack, 3–10% of cross sectional area affected), 7 (moderate/severe attack, penetration, 10–30% of cross sectional area affected), 6 (severe attack, 30–50% of cross sectional area affected), 4 (very severe attack, 50–70% of cross sectional area affected) or 0 (failure)).

**Bamboo Species**	**Mean Visual Rating**	**Mean Mass Loss (g)**	**Mean Percent Mass loss (%)**	**Mean Percent Termite Mortality (%)**
*Guadua angustifolia* (GA)	5.20	0.6514	24.52	19.30
	(±1.10)	(±0.04)a	(±3.28)	(±3.52)d
*Bambusa hirose* (BH)	6.00	0.5700	20.97	41.00
	(±1.41)	(±0.10)ab ^a^	(±8.02)	(±13.98)abc
*Dendrocalamus latiflorus* (DL)	6.00	0.6068	21.04	28.10
	(±0.00)	(±0.06)a	(±2.64)	(±5.79)acd
*Dendrocalamus brandisii* (DB)	7.00	0.4665	16.76	31.50
	(±0.00)	(±0.014)bd	(±2.23)	(±2.79)acd
*Bambusa oldhamii* (BO)	6.40	0.4526	15.73	49.60
	(±0.55)	(±0.10)bc	(±2.87)	(±9.83)b
*Gigantocholoa pseudoarundinacea* (GP)	7.40	0.3928	12.96	41.50
	(±0.55)	(±0.03)cd	(±1.24)	(±9.07)abc

**Table 3:** Summary of results (Two-way ANOVA, Ryan-Einot-Gabriel-Welsch Multiple Range Test (REGWQ), SAS 9.2).

		**Mean Mass Loss (g)**
**Wood Species (*p*<0.0001)**	*Bambusa hirose* (**BH**)	0.5957a
	*B. oldhamii* (**BO)**	0.4682bc
	*Dendrocalamus brandisii* (**DB)**	0.4946b
	*D. latiflorus* (**DL**)	0.5906a
	*Gigantocholoa pseudoarundinacea* (**GP**)	0.4114c
	*Guadua angustifolia* (**GA**)	0.6713a

**Termite Species (*p* = 0.0855**)	*Coptotermes formosanus*	0.5539a
	*Coptotermes gestroi*	0.5234a

**Table 4:** Different resistance levels of bamboo species.

**Bamboo species**	**Resistance class**
*Guadua angustifolia*	Susceptible
*Bambusa hirose*	Susceptible
*Dendrocalamus latiflorus*	Susceptible
*D. brandisii*	Slightly resistance
*B. oldhamii*	Slightly resistance
*Gigantocholoa pseudoarundinacea*	Slightly resistance
